# The Role of HCV E2 Protein Glycosylation in Functioning of Virus Envelope Proteins in Insect and Mammalian Cells

**Published:** 2015

**Authors:** O. V. Orlova, V. L. Drutsa, P. V. Spirin, V. S. Prasolov, P. M. Rubtsov, S. N. Kochetkov, S. N. Beljelarskaya

**Affiliations:** Engelhardt Institute of Molecular Biology, Russian Academy of Sciences, Vavilova Str., 32, 119991, Moscow, Russia; Chemical Department of Moscow State University, Leninskie Gory, 1, Bld. 3, 119899, Moscow, Russia

**Keywords:** baculovirus expression vector system, hepatitis C virus envelope proteins E1 and E2, virus-like particles, N-linked protein glycosylation, Sf9 insect cells, mammalian HEK293T and Huh7.0 cells, oligonucleotide-directed mutagenesis

## Abstract

The hepatitis C virus (HCV) envelope proteins E1 and E2, being virion components, are involved in the formation of infectious particles in infected cells. The detailed structure of the infectious particle of HCV remains poorly understood. Moreover, the virion assembly and release of virions by the cell are the least understood processes. It is believed that virion properties depend on glycosylation of the virus envelope proteins in a cell, while glycansat several glycosylation sites of these proteins play a pivotal role in protein functioning and the HCV life cycle. N-glycans of glycoproteins can influence viral particle formation, virus binding to cell surface, and HCV pathogenesis. We studied the effect of glycans on the folding ofthe E2 glycoprotein, formation of functional glycoprotein complexes and virus particles in insect and mammalian cells. In order to investigate these processes, point mutations of the N-glycosylation sites of HCV protein E2 (genotype 1b strain 274933RU) were generated and the mutant proteins were further analyzed in the baculovirus expression system. Elimination of the single glycosylation sites of the E2 glycoprotein, except for the N6 site, did not affect its synthesis efficiency in Sf9 insect cells, while the electrophoretic mobility of mutant proteins increased in proportion to the decrease in the number of glycosylation sites. The level of synthesis of HCV glycoprotein E2 in human HEK293T cells depended on the presence of glycans at the N1 and N8 glycosylation sites in contrast to Sf9 cells. At the same time, elimination of glycans at the N1, N2, and N10 sites led to the accumulation of unproductive E1E2 dimers as aggregates and productive assembly suppression of virus-like particles both in insect and mammalian cells. In addition, elimination of single glycosylation sites of HCV E2 had no impact on the RNA synthesis of structural proteins and formation of virus-like particles in insect and mammalian cells.

## INTRODUCTION

Hepatitis C virus is one of the most nefarious pathogens causing severe liver diseases, including cirrhosis and hepatocellular carcinoma. The range of drugs for the HCV infection is rather limited, while immunoprophylaxis of the HCV infection is not yet available. The high replication activity of HCV with the lack of proofreading ability of the viral RNA-dependentRNA polymerase results in a high genetic variability of the virus. As a consequence,HCV circulates in the serum of an infected person as a population of quasi species differing in their genomes by1–5%. Distinct strains of the same HCV subtype can differ in their nucleotide sequence by 5–15%; subtypes, by 10–30%; and different genotypes, by 30–50% [1]. Some strains have higher virulence; yet,definite molecular determinants for such phenotype still remain unknown. Hepatocytes are the main cellular target of HCV. Binding of a virus particle to the cell surface remains poorly studied. Furthermore, not all receptors, including HCV-specific ones, are known.

Virus envelope glycoproteins affect the binding of a viral particle to hepatocyte receptors and its absorption by a cell. The mechanism of assembly of viral structural proteins and RNA into new viral particles, along with the routes of virus transmission into a cell, remains poorly studied [2]. HCV is the only member of the genus *Hepacivirus *and belongs to the *Flaviviridae *family. Its genome encodes a single polyprotein precursor. Structural and nonstructural viral proteins are formed under the effect of cellular and viral proteases [3-5]. The capsid protein C and envelope proteins E1 and E2 are structural proteins. Envelope proteins undergo post-translational modification such as N-glycosylation, wherein the unbranched oligosaccharide chain consisting of nine mannose residues (Man) and three glycose residues (Glc) are bound to a specific asparagine residue as a part of the Asn-X-Ser or Asn-X-Thr sequence (where X is any amino acid except proline) [6, 7]. The glycoproteins of the viral envelope are heavily glycosylated. The degree of conservation of the glycosylation sites 9–11 in E2 and 4–5 in E1 is high, which is an indicationthat they play an important role in the functioning of these proteins in the life cycle of HCV [8]. It should be noted that it still remains unknown what the actual number of glycosylation sites of proteins is; exactly which sites participate in protein modification; and whether all potential sites undergo glycosylation* in vivo*.

The nature of glycoprotein glycosylation plays an important role in its functioning. Thus, glycoprotein E2 can be the receptor binding subunit of the HCV envelope. It has been shown that, depending on theHCV strain,a number of E2 glycans can determine the possibility of penetration of the virus into a cell, allowing the E2 glycoprotein to interact with cellular receptors. Certain E2 glycans are involved in the modulation of the immune response. It is thought that the glycans associated with the viral envelope influence protein folding, with the involvement of the chaperones of the endoplasmic reticulum (ER) and the productive assembly of the viral particles that are able to penetrate and infect another cell. Oligosaccharide attachment to a protein is related to its folding, while the glycoprotein penetrates the calnexin/calreticulin cycle interacting specifically with the endoplasmic reticulum chaperones that ensure its partial folding. Binding of glycoproteins to chaperones and their dissociation are followed by the detachment (trimming) of abundant glycose and mannose residues and reglycosylation of N-linked glycans.

Envelope protein E2 accumulates in the ER lumen bothas a properly folded glycoprotein and aggregates of misfolded proteins. A portion of E2 remains unglycosylated in the cytosol and is degraded via the ubiquitin– proteasome pathway after ubiquitination. The calnexin protein interacts with noncovalently bound E1E2 complexes, while calreticulin interacts with aggregates of the misfolded proteins [9]. The first type of proteins provides binding of the virus to cellular receptors and penetration of the viral particle into a cell; it also influences the formation of its antigene composition and probably plays an essential role in viral pathogenesis [10]. The formation of misfolded glycoproteins aggregates can lead to the emergence of defective viral particles incapable of binding to new cells [11-13]. The HCV envelope proteins can also influence each other’s folding. Thus, E2 acts as a chaperone during E1 folding and the formation of functional heterodimers, although the E1 protein also assists the productive folding of E2 [14, 15]. Assembly of HCV virions remains insufficiently studied due to the absence of appropriate cell models that would allow one to obtain infectious virus particles. The role of glycans in the functioning of virus envelope proteins of different genotypes in an infected cell also remains poorly investigated.

In this study, we investigated the influence of N-glycans of the E2 protein of HCV (genotype 1b strain 274933RU [16]) on the synthesis of structural proteinsand formation of virus particles in Sf9 insect cells and HEK293T human cells transfected with baculoviruses vectors directing the gene expression of HCV structural proteins [17].

## MATERIALS AND METHODS

**Bacterial strains, cells, and plasmids**

*Escherichia coli, Spodopterafrugiperda Sf9 *cells, and mammalian HEK293T and Huh7.0 cells were used. *Escherichia coli *cells of the DH5a and DH10Bac strains were transformed with the plasmid DNA according to the manufacturer’s recommendation (Amersham, USA). Isolation and purification of plasmids, restriction enzyme digestion, ligation, agarose gel electrophoresis of DNA, and other genetic engineering experiments were carried out using the standard protocols [18].

Sf9 insect cells were cultured at 27°C in a Sf-900 II medium supplemented with 10% fetal bovine serum following the basic procedures that were previously discussed and described in the protocol [19]. To assess virus titer, amplification of recombinant virus, infection of Sf9 cells with recombinant baculovirus, and viral gene expression using the same protocol were used.

Human embryonic kidney cells (HEK293T cell line) were cultured at 37°C and 5% CO_2_ in DMEM media supplemented with 10% fetal bovine serum, 4 mM*L*-glutamine, 1 mM sodium pyruvate, and streptomycin/ penicillin at concentrations of 100 mg/ml and 100 IU/ml, respectively.

Recombinant constructs for the corresponding cDNA fragments of the genes of HCV structural proteins, recombinant bacmids, as well as recombinant baculovirus bv-CE1E2, were obtained and analyzed according to the previously discussed procedures [20].

**Site-directed mutagenesis**

The DNA fragment corresponding to cDNA of the gene encoding HCV glycoprotein E2 was cloned into plasmid pFastBacHTb at NcoI–EcoRI restriction sites according to the standard protocol [20]. Oligonucleotide primers were constructed to obtain a series of recombinant plasmids bearing cDNA of the E2 protein with mutations of glycosylation sites (*Table*). Each primer consisted of 25–30 nucleotides and contained the sequence encoding the *N*-glycosylation site: Asn-X-Thr/Ser (X^1^Pro), in which the triplet encoding Asn was substituted with the triplet encoding Gln.

**Table:** Primers used

Primer	Orientation	Nucleotide sequence 5’ → 3’
28-E2N1m	-	TG AAT ACC CAA GGC AGC TGG CAC AT
30-E2N2m	-	TGG CAC ATC CAA AGT ACT GCC CTA AAT TGC
30-E2N3m	-	GCCCTAAATTGCCAAGACTCCCTCCAAACT
30-E2N4m	-	GCA CAC AAG TTC CAA TCG TCC GGG TGC CCG
25-E2N6m	-	TGG GGG GAG CAA GAG ACA GAC GTG A
30-E2N7m	-	GTG ATG CTC CTC CAA AAC ACG CGT CCG CCA
30-E2N8m	-	TGT ACA TGG ATG CAA AGT ACT GGG TTC ACT
27-E2N9m	-	GGGGTCGGTCAACGCACCTTGATTTGC
30-E2N10m	-	TAC CCC TGC ACT CTC CAA TTT TTC CAT CAT
27-E2N11m	-	GCCGCATGCCAATGGACTCGAGGAGAGCGC
E2 for	+	AGGTCTAGAATGTTATGATTGTTTTGCTAC
E2 Back	+	CT ATA GTG TCA CCT AAA TCC GAA AGC TTC GGC CTC AGC TTG AG

The method described by Drutsa*et al.*was used for mutagenesis [21]. PCR was performed on a CycloTemp 107 programmable thermocycler (ResursPribor, Russia). Predetermined base substitutions were verified by sequencing.

**Analysis of total cellular DNA**

The total cellular DNAwas isolated from insect cells 72 h after infection with recombinant baculoviruses bv- CE1E2, bv-E2mut, bv-E1E2mut, bv-CE1E2mut (multiplicity of infection being 5 CFU per cell) [20]. The presence of cDNA genes of HCV structural proteins in the total cellular DNA was assessed by PCR using the baculovirus primers of vector pFastBacHT (the forward 5'-GTGGTTGGCTACGTATACTCC-3'and reverse 5'-CCTCTACAAATGTGGTATGGC-3').

**Analysis of HCV RNA using RT-PCR**

Sf9 cells were infected with recombinant baculoviruses bv-CE1E2mut (5 CFU per cell) and incubated at 27°C for 72 h. After 72 h, the medium was eliminated and cell debris was removed by low-speed centrifugation. The supernatant was centrifuged over a cushion of 30% sucrose at 23,000 *g *for 16 h at 4°C (Becman Coulter Optima L-100XP centrifuge, 80Ti rotor). RNA extraction with TRIzol (Invitrogen) was performed according to the manufacturer’s recommendations, and RNA was then treated with DNase I (Promega). Reverse transcription was carried out using a Phusion RT-PCR Kit (Thermo Scientific). The obtained cDNA was amplified using PCR with primers of the genes of structural and non-structural HCV proteins. The total cellular RNA was obtained from Sf9 cells infected with recombinant baculoviruses bv-CE1E2mut (5 CFU per cell) that was incubated at 27°C for 72 h and then washed three times with phosphate buffered saline (PBS). RNA isolation, reverse transcription, and amplification were performed using the previously mentioned protocols.

HEK283T cells were transfected with recombinant plasmids BacMamCE1E2mut-GFP (5 CFU per cell) and incubated at 37°C for 48 h. The medium was removed, RNA was isolated, and reverse transcription and amplification were performed using the previously mentioned protocols.

**Anti-HCV Antibodies**

Mouse monoclonal antibodies to the HCV E1 (Hep C E1 1879: sc-65459) and HCV E2 (Hep C E2 BDI167: sc- 57769) (SantaCruz Biotechnology, USA) proteins, as well as monoclonal antibodies to calnexin (AF18) and calreticulin(FMC75) (Abcam, UK), were used. Polyclonal rabbit antibodies to the structural protein C were kindly provided by M.G. Isagulyants(Ivanovsky Institute of Virology,Russian Academy of Sciences, Moscow). Anti-mouse IgG antibodies (AB6706-1EA) conjugated to horseradish peroxidase (Sigma, USA) were used as secondary antibodies.

**Western blotting and immunoprecipitation**

After 72 h of infection with the recombinant baculovirusesbv- E2mut, bv-E1E2mut, bv-CE1E2mut (multiplicity of infection of 5 CFU per cell), Sf9 cells were harvested, washed three times with PBS (1.47 mM KH_2_PO_4_, 4.29 mM Na_2_HPO_4_·7H_2_O, 137 mMNaCl, 2.68 mMKCl), resuspended in a TNC lysis buffer containing 0.25% digitonin, and disrupted using ultrasonic vibrations. Cell debris was removed by centrifugation (15,000 *g*, 15 min, 4°C). Cell lysate was loaded into a 12% denaturing gel (each sample contained 10 μg of the protein). After electrophoresis, the proteins were transferred onto a Hybond-ECL nitrocellulose membrane (Amersham Biosciences, USA) using semi-dry electrophoretic transfer. The membranes were washed with PBS containing 5% nonfat dry milk, incubated with primary antibodies to the structural HCV proteins E1 or E2 (dilution 1 : 1500 for E1 and 1 : 2000 for E2), to calnexin or calreticulin (dilutions 1 : 1000 and 1 : 2000, respectively), and then to secondary antibodies (dilution 1 : 20,000). Protein complexes on immunoblots were detected using ECL and ECL Plus chemiluminescent reagents (Western blotting detection reagents and analysis systems, Amersham Biosciences) according to the manufacturer’s recommendations.

For immunoprecipitation, the cells infected with recombinant baculoviruses bv-E2mut, bv-E1E2mut, and bv-CE1E2mut were harvested after 72 h of infection; the cells were lysed, and subsequently cell debris and nuclei were removed. The structural proteins and their complexes were precipitated by monoclonal antibodies to HCV E1 and HCV E2, calnexin and calreticulin in dilution 1 : 1000 (according to the manufacturer’s recommendations). The precipitated proteins were separated using PAGE (12% denaturing gel), transferred onto a nitrocellulose membrane, and incubated with primary antibodies in the previously mentioned dilutions; the membranes were treated with secondary antibodies.

**Analysis of glycosylation by treatment with endoglycosidase H (Endo H)**

The proteins of the cell lysate were incubated with the corresponding monoclonal antibodies at 4°C. The obtained complex was precipitated by protein G sepharose (BioVision, USA). 1 μl of a 10× denaturing buffer (5% SDS, 0.4 M DTT) was added to the precipitated protein, the mixture volume was then diluted with water to 10 μl, and the mixture was boiled for 10 min. Next, the mixture volume was diluted to 20 μl by adding 2 μl of a 10× G5 reaction buffer (50 mM sodium citrate), 3 μl of water, and 5 μl of a Endo H solution (5 units) (P0702S BioLabs Inc., UK). The mixture was incubated for 15 min at 37°C and analyzed by PAGE (12% denaturing gel).

**Preparation and purification of virus-like particles (VLPs)**

A cell monolayer cultured at 27°C was infected with recombinant baculovirus bv-CE1E2 (10 CFU per cell). After 72 h, the cells (2 × 10^8^) were harvested, washed three times with PBS, re-suspended in a TNC lysis buffer (10 mMTris-HCl, pH 7.5, 150 mMNaCl, 1 mM CaCl_2_, 1 mM PMSF, protease inhibitors cocktail II (Calbiochem, USA) (1 : 200) containing 0.25% digitonin, and disrupted using ultrasonic vibrations. Cell debris was removed from the VLPs extracted from homogenized lysates by low-speed centrifugation (1,200 *g*, 15 min, 4°C). After purification,VLPs were concentrated by centrifugation over a cushion of 30% sucrose at 23,000* g *for 16 h at 4°C. The VLPs precipitate was re-suspended in a TNC buffer containing 1 mM PMSF, protease inhibitors (1 : 200), and analyzed using centrifugation in a sucrose gradient.

**Centrifugation in sucrose gradien**

The VLPs precipitate re-suspended in a 100 μl TNC buffer containing 1 mM PMSF and protease inhibitors (1 : 200) was placed layer after layer on sucrose solutions of different concentrations (from 10 to 60% in 50 mMTris-HCl, 100 mMNaCl, pH 7.4) and centrifuged at 200,000 *g *for 2.5 h at 4°C (Becman Coulter Optima L-100XP centrifuge, 80Ti rotor). Ten 1 ml fractions were collected, and each fraction was concentrated by high-speed ultracentrifugation at 230,000 *g *for 16 h at 4°C. The precipitate was dissolved in a 100 μl TNC buffer containing 1 mM PMSF and protease inhibitors (1 : 200) and analyzed using Western blotting [18, 22].

**Analysis of VLP binding to CD81 receptor**

Huh7.0 cells were incubated in the presence of VLPs obtained in Sf9 cells for 1 h at 4°C. Huh7.0 cells pre-incubated with anti-CD81 antibodies(20 μg/ml, 1 h at 4°C) to block the CD81 receptor were used as a control. The cells were harvested, washed twice with PBS to remove unboundVLPs, and analyzed using Westernblotting with anti-E2 antibodies.

**Fluorescent microscopy and flow cytometry**

HEK293T cells were trypsinized after 48 h of transfection with the recombinant plasmids BacMam- CE1E2mutGFP. The cells were collected, washed twice with PBS, and analyzed using flow cytofluorometry( Beckman Coulter EPICS, USA) and Western blotting.

## RESULTS AND DISCUSSION

**Preparation of genetic engineering constructs and site-directed mutagenesis**

We had previously studied the influence of *N*-glycans of HCV protein E1 on its folding and productive heterodimer assembly of E1E2 in insect and mammalian cells. We have revealed that glycans linked to the N1 and N5 sites of the E1 protein play the most significant role in the proper folding of these proteins [23]. In this study, we have investigated the involvement of glycans of the HCV glycoprotein E2 (genotype 1b strain 274933RU [16]) in the glycoprotein foldingand formation of functional glycoprotein complexes and virus particles in insect and mammalian cells. For this purpose, we generated single-point mutations in the E2 at* N*-glycosylation sites and expressed the genes of mutant proteins in insect and mammalian cells using the baculovirus expression system [17, 20].

**Fig. 1:**
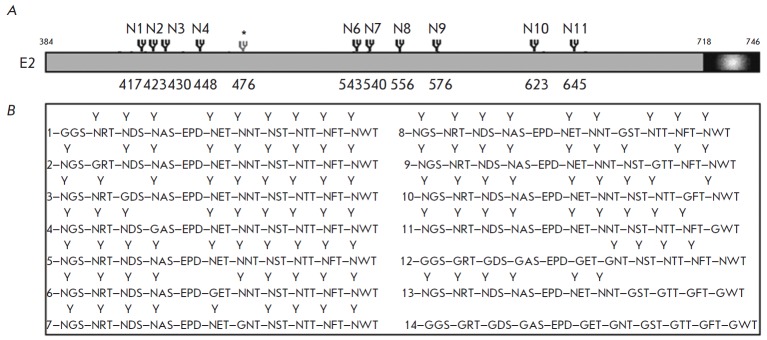
N-glycosylation sites of the HCV E2structural protein and its mutant variants. A – Positions of N1–N11 glycosylation sites in the polypeptide chain of E2. B – Mutant variants of glycoprotein E2 with modified (disrupted) glycosylation sites: 1, N1; 2, N2; 3, N3; 4, N4; 5, wild-type E2; 6, N6; 7, N7; 8, N8; 9, N9; 10, N10; 11, N11; 12, N1–N7(mL); 13, N8– N11(mR); 14, N1–N11(ΣN). Glycosylation sites are marked with “Y”

The DNA fragment corresponding to the cDNA sequence of the gene encoding the HCV glycoprotein E2 was cloned into the pFastBacHTb plasmid at NcoI–EcoRI using the standard protocol [20]. Oligonucleotide primers were constructed according to Drutsa *et al. *to obtain a series of recombinant plasmids bearing cDNA of the E2 protein with point mutations at glycosylation sites [20, 21] (*see *Materials and Methods section).The presence of all predetermined base substitutions was verified by sequencing. As a result, we obtained vector DNA pFastBacHTbE- 2mut containing E2 genes with the generated mutations, which were subsequently used to construct the pFastBacHTbE1E2mut, pFastBacHTbCE1E2mut, and BacMamCE1E2mutGFP vectors. The scheme of potential positions of N-glycosylation sites in the HCV E2 protein and the constructed mutant variant of E2 are shown in *Fig. 1*.

## INVESTIGATION OF THE ROLE OF THE GLYCOSYLATION OF HCV PROTEIN E2 IN THE FUNCTIONING OF VIRUS ENVELOPE PROTEINS ININSECT AND MAMMALIAN CELLS

**Influence of N-glycans of HCV glycoprotein E2 on the expression of the genes of mutant HCV proteins E2 in insect and mammalian cells**

**Fig. 2:**
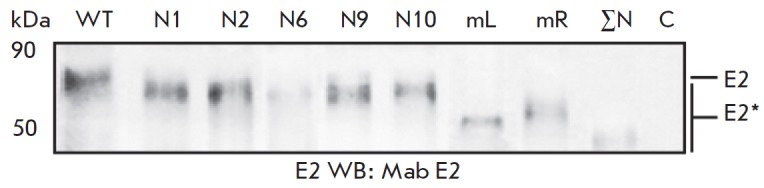
Analysis of the gene expression of the mutant E2 proteins of HCV in Sf9 cells.Western blotting using anti-E2 antibodies following PAGE in 12% denaturing gel. Lysates of cells infected with recombinant baculoviruses: wild-type E2 (WT) and E2 with mutations of the glycosylationsites N1; N2;N6; N9; N10; N1–N7(mL); N8–N11 (mR); N1–N11(ΣN; mutations of all sites); C – negative control (Hsp90). Here and in Figs. 3–9, numbers on the left side are the protein molecular weight marker, kDa. Mutant proteins are marked as E2*

We have previously shown that effective posttranslational glycosylation of HCV envelope proteins occurs in insect cells [22]. We have also revealed that disruption of the glycosylation sites of HCV glycoprotein E1 in various combinations does not influence its synthesis in Sf9 cells, although the absence of carbohydrate chains at the N1 and N5 E1 sites drastically reduces the level of its expression in HEK293T mammalian cells [23]. An analysis of the gene expression of mutant E2 proteins in Sf9 insect cells revealed that disruption of glycosylation sites in various combinations does not influence E2 synthesis, while electrophoretic mobility of mutant proteins increases in proportion to the decrease in the number of glycosylation sites (*Fig. 2*).

**Fig. 3:**
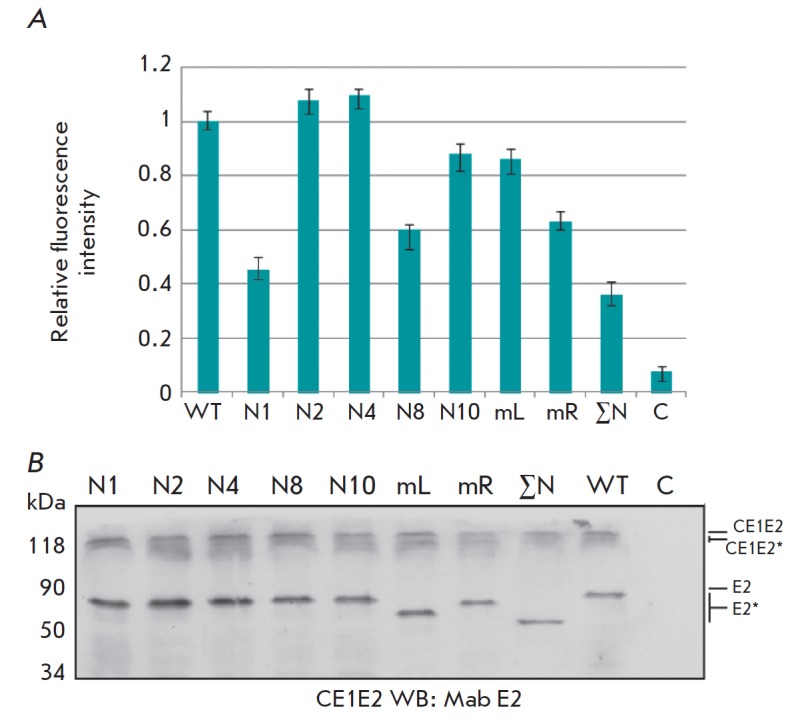
Analysis of the gene expression of mutant E2 proteinswithinthe HCV CE1E2polypeptide in mammalian cells. A – Flow cytofluorometry of HEK293T cells transfected with the pFastBacMam CE1E2mutGFP plasmid. Relative values of fluorescence intensity are plotted on the Y axis,and E2 variants with mutations at the glycosylation sites N1, N2, N4, N8 N10, mL(N1–N7), mR (N8–N11), and ΣN(N1–N11) are indicated along the X axis; (B) PAGE in 8% denaturing gel and Western blotting of lysates of HEK293T cells transfected with pFastBacMamCE1E2GFP and synthesizing the following variants of E2: wild-type E2 (WT) and E2 with mutations of N1, N2, N4, N8, N10, N1N7(mL), N8–N11 (mR), and N1–N11(ΣN, mutations of all E2 glycosylation sites) glycosylation sites using anti-E2 antibodies; C – negative control (Hsp90). Mutant proteins are marked as E2*

The intensity of the synthesis of HCV E2 in mammalian cells was found to depend on the presence of glycans at the specific glycosylation sites. To analyze the influence of N-glycans of HCV glycoprotein E2 on the efficiency of the expression of HCV envelope proteins in mammalian cells, plasmids pFastBacMam1GFP based on a baculovirus vector with expression cassettes under the control of the cytomegalovirus promoter (CMV) carrying the cDNA of mutant E2 were constructed. Human cells HEK293T were transfected with the resulting vector DNA pFastBacMam-CE1E2mut- GFP encoding E2 with point mutations at the glycosylation sites N1, N2, N4, N8, N10, mL(N1–N7), mR(N8– N11),and ΣN(N1–N11). Expression of the genes of mutant E2 proteins of HCV and the efficiency of their glycosylation in cells were assessed according to the level of synthesis of polypeptides CE1E2mutGFP using flow cytofluorometry and PAGE, followed by immunoblotting (*Fig. 3A,B*).

According to flow cytofluorometry data, the absence of N1 and N8 glycosylation sites in HCV E2 significantly reduces GFP fluorescence, which attests to a decreased E2 synthesis in CE1E2mutGFP polypeptides in HEK293T cells compared to the control cells. The mutation at the N10 site leads to an insignificant decrease in the synthesis of E2 glycoprotein. PAGE followed by immunoblotting revealed that mutant E2 variants were synthesized in mammalian cells and that the intensity of their synthesis depends on the presence of glycans at specific glycosylation sites of a protein. In addition, electrophoretic mobility of the proteins increased in proportion to the decrease in the number of glycosylation sites.

**Fig. 4:**
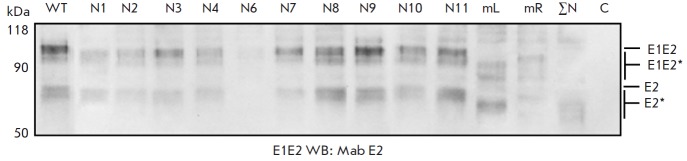
Analysis of the gene expression of mutant E2 proteins as part of the HCV E1E2 polypeptide in Sf9 cells. Western Blotting using anti-E2 antibodies following PAGE in 10% denaturing gel. Lysates of cells infected with recombinant baculovirus synthesizing E2 as part of E1E2: wild-type E2 (WT) and E2 with mutations of the glycosylation sites N1; N2; N3; N4; N6; N7; N8; N9; N10; N11; N1–N7(mL); N8–N11(mR); N1–N11(ΣN; mutations of all glycosylation sites); C – negative control (Hsp90). Mutant proteins are marked as E2*

An analysis of the expression of the genes of mutant E2 proteins of HCV in E1E2 has revealed that the absence of glycans at any site except for N6 does not influence their synthesis in Sf9 cells (*Fig. 4*).

Treatment of mutant HCV glycoproteins E2 with endoglycosidase H (Endo H) followed by Westernblotting has shown that mutant variants of the glycoprotein are sensitive to endoglycosidase activity (data are not presented), while glycosylation of the synthesized mutant glycoproteins occurs in insect cells.

**Influence of N-glycans of HCV glycoprotein E2 on the formation of a productive E1E2 complex in insect cells**

**Fig. 5:**
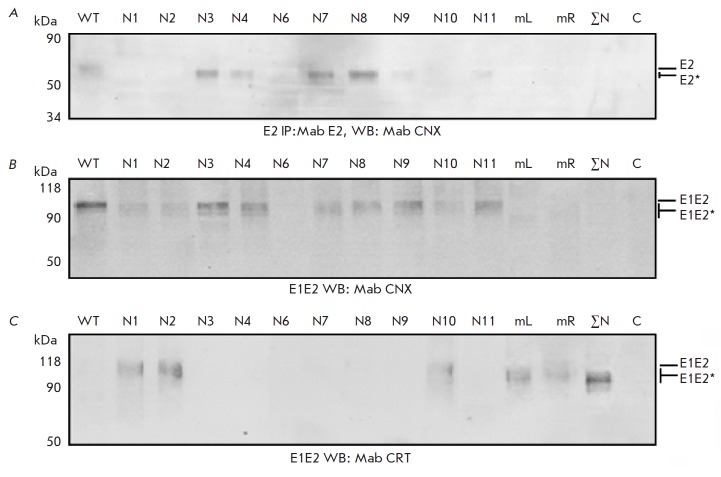
Analysis of the gene expression of mutant E2 proteins in Sf9 cells.A – Western blotting with anti-calnexin antibodies following PAGE in 12% denaturing gel after preliminary immunoprecipitation with anti-E2 antibodies. Lysates of cells infected with recombinant baculovirus producing wild-type E2 (WT) and E2 with mutations of the glycosylation sites N1; N2; N3; N4; N6; N7; N8; N9; N10; N11; N1–N7(mL); N8–N11 (mR); N1–N11(ΣN;mutations of all E2 glycosylation sites). Western blotting using antibodies to (B) calnexin and (C) calreticulin following PAGE in 10% denaturing gel. Lysates of cells infected with recombinant baculovirus synthesizing mutant E2 as part of E1E2. C –negative control (Hsp90). Mutant proteins are marked as E2*

We have shown that the assembly of HCV glycoprotein complexes E1E2 in insect cells depends on the disorder of the N1 and N5 glycosylation sites of glycoprotein E1, while mutations in other sites do not influence the assembly [23]. In this study, the influence of the disorder of glycosylation sites in E2 on its folding and formation of E1E2 heterodimers in insect cells, identically to the case of mutant glycoprotein E1, was estimated by their interaction with calnexin and calreticulin (*Fig. 5 A-C*).

An analysis of the expression of the genes of mutant HCV proteinsE2 inthe glycoprotein complexes E1E2 in Sf9 insect cells has revealed that the noncovalently bound E1E2 complex is formed as in the case of the expression of wild-type E2 if one of the glycosylation sites is disrupted (N3, N4, N7, or N8). Mutant E2 containing neither the N9 nor N10 site have a moderate effect on the assembly of HCV envelope glycoproteins. The interaction between heterodimers and calnexinis enhanced, and the assembly of the productive E1E2 complex is disrupted as the number of damaged sites (N1–N7(mL) and N8–N11(mR)) decreases. Aggregates of misfolded E1E2 dimers formed by the E2 protein with mutations at all glycosylation sites do not interact with calnexin. Interestingly, the assembly of the noncovalently bound E1E2 complex is also disrupted when one of the sites (N1, N2, or N10) is damaged. Mutations at these glycosylation sites in E2 apparently interfere with the formation of a properconformation of the proteins forming the functional E1E2 complex.

**Influence of N-glycans of HCV glycoprotein E2 on the formation of virus-like particles in Sf9 and HEK293T cells**

**Fig. 6:**
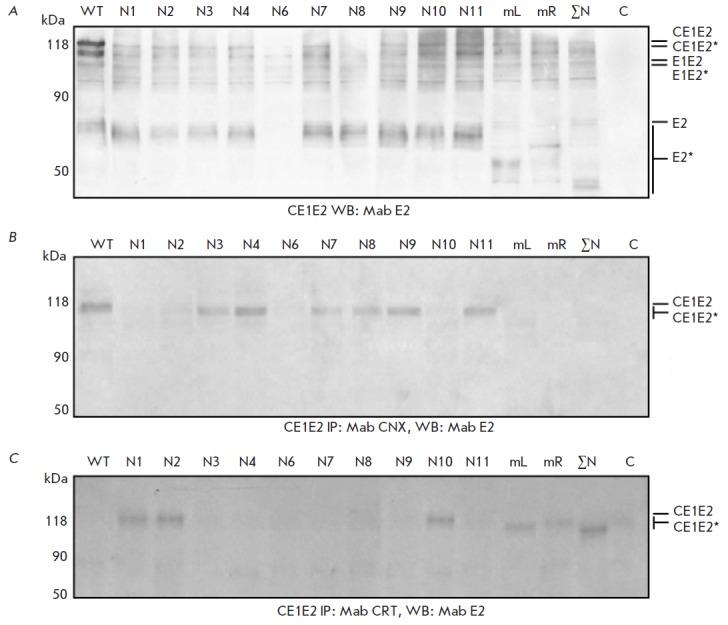
Analysis of the gene expression of mutant E2 proteins as part of HCV CE1E2 in Sf9 cells. PAGE in 12% denaturing gel followed by Western blotting using anti-E2 antibodies (A) and anti-E2 antibodies after preliminary immunoprecipitation with antibodies to calnexin (B) and calreticulin (C). Lysates of cells infected with recombinant baculovirus synthesizing E2 as part of HCV CE1E2: wild-type E2 (WT) and E2 with mutations at the glycosylation sites N1; N2; N3; N4; N6; N7; N8; N9; N10; N11; N1– N7(mL); N8–N11 (mR); N1–N11(ΣN;mutations of all E2 glycosylation sites). C –negative control (Hsp90). Mutant proteins are marked as E2*

It was shown that the synthesis of the structural proteins C (core), E1, and E2 of HCV in insect cells is accompanied by the formation of virus-like particles. We have previously shown that the recombinant structural proteins of HCV (including mutant E1 protein) synthesized in insect cells are incorporated into the ER membranes where their folding, generation of E1E2, and formation of VLPs occur. Formation of VLPs in microsomal fractions with Sf9 insect cells infected with recombinant baculoviruses having been removed was detected using electron microscopy [20]. We revealed that the absence of glycans at the glycosylation sites of the E1 protein does not influence the formation of VLPs in insect cells [23]. An analysis of the expression of the genes of mutant E2 proteins of HCV as part of CE1E2 in Sf9 insect cells showed that disruption of glycosylation sites in various combinations (except for the N6 site) has no effect on their synthesis, while the electrophoretic mobility of mutant proteins increases in proportion to the reduction in the number of glycosylation sites (*Fig. 6A*).

**Fig. 7:**
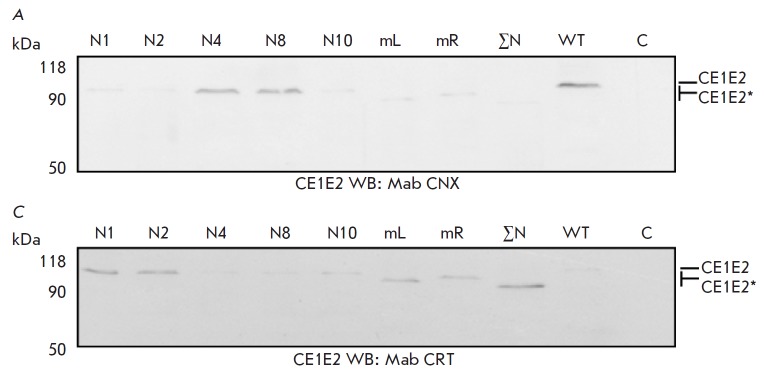
Analysis of the gene expression of mutant E2 proteins as part of HCV CE1E2 in mammalian HEK293T cells. Western blotting using antibodies to calnexin (A) and calreticulin (B) following PAGE in 10% denaturing gel. Lysates of cells infected with recombinant plasmid DNA pFastBacMam CE1E2GFP synthesizing E2 with mutations at the glycosylation sites N1; N2; N4; N8; N10; N1–N7(mL); N8–N11 (mR); and N1–N11(ΣN; mutations of all E2 glycosylation sites). WT – wild-type E2; C – negative control (Hsp90). Mutant proteins are marked as E2*

Mutations introduced at the N3, N4, N7, N8, N9, N11 glycosylation sites of E2 do not interfere with glycoprotein folding as part of HCV CE1E2 in Sf9 cells. Meanwhile, the modification of the N1, N2, and N10 sites disrupts the assembly, leading to the formation of unproductive E1E2 heterodimers and their misfoldingin VLPs (*Fig. 6 B,C*). The formation of misfolded glycoprotein aggregates does not interfere with the formation of HCV VLPs in insect cells, but apparently it leads to the formation of defective virus particles that differ from natural ones. An analysis of the expression of the genes of HCV mutant E2 proteins in CE1E2 in both HEK293T and Sf9 cells showed that proper folding of glycoproteins in HCV CE1E2 is not disrupted upon formation of the E2 protein without either the N4 or N8 glycosylation site. In addition, the interaction between the resulting heterodimers and calnexin decreases, while the interaction with calreticulin increases with a rising number of damaged N1–N7(mL) or N8–N11(mR) glycosylation sites in E2. The dimers formed by E2 with mutations at all glycosylation sites (ΣN) interact with calreticulin. Interestingly, the assembly of the productive E1E2 complex and its folding in VLPs are disrupted as a result of the damage to the E2 N1, N2, and partially N10 glycosylation sites both in mammalian and insect cells (*Fig. 7 A,B*).

The absence of carbohydrate chains at thee N1, N2 sites and,to a smaller extent, at the N10 site of the E2 glycoproteinseems, play an essential role in the misfolding of the proteins of the HCV VLP functional complex, thus impeding the formation of mature virus particles [24].

**Fig. 8:**
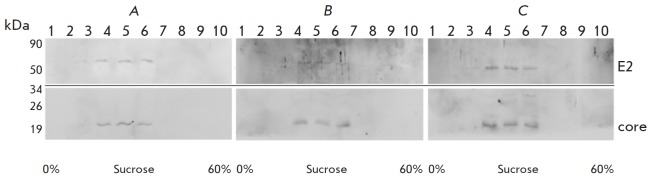
Analysis of HCV-like particles isolated from HEK293T cells by centrifugation in sucrose gradient. Western blotting of ten VLP fractions, top-to-bottom, with antibodies to HCV core and E2 proteins following PAGE in 10% denaturing gel. HEK293T cells were transfected with recombinant DNA pFastBacMamCE1E2-GFP: intact DNA (A) andDNA with mutations at the glycosylation siteN1of E2 (B) or in all E2 glycosylation sites (C)

We have previously demonstrated the formation of HCV VLPs in insect cells using Western blotting with anti-HCV antibodies and electron microscopy. To determine the biophysical characteristics of HCV VLPs obtained in mammalian cells, VLPs were purified and concentrated by centrifugation over a cushion of 30% sucrose at 23,000 *g*. The VLP precipitate was then analyzed by sucrose gradient centrifugation as described in the *Materials and Methods *section. The collected fractions were analyzed by Western blotting using antibodies to the structural proteins (*Fig. 8 A-C*).

Immunoblotting using antibodies to the proteins C and E2 revealed that VLPs are contained in all fractions at a density of 1.14–1.16 g/cm^3^. This fact can be an indication that RNA fragments are present in VLPs [25].

RT-PCR on total RNA isolated from insect cells infected with recombinant baculovirus bv-CE1E2mut with primers for the genes of the HCV proteins C and E2 revealed fragments of structural proteins (data are not presented). Similar results were obtained for HEK293T cells. Thus, N-glycans of HCV glycoprotein E2 do not influence the synthesis of RNA of structural proteins in insect and mammalian cells and probably do not affect the incorporation of these RNA into virus- like particles.

**Influence of CD81 receptor on binding of recombinant HCV VLPs to Huh7.0 hepatoma cells**

**Fig. 9:**
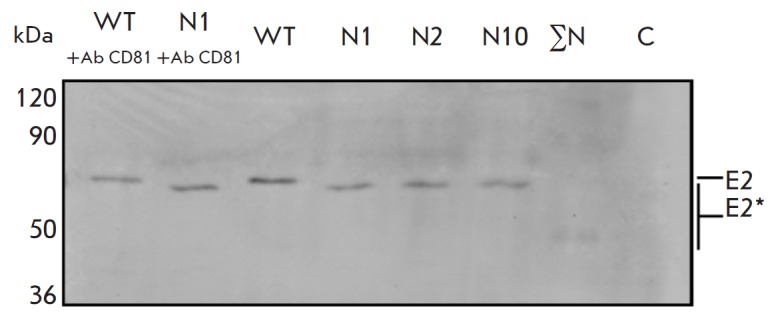
Binding of mutant E2 proteins within recombinant HCV-like particles to Huh-7 cells, either treated with anti- CD81 antibodies or not. Western blotting of mutant E2 proteins using anti-E2 antibodies following PAGE in 10% denaturing gel. Lysates of Huh-7 cells after incubation with VLPs isolated from Sf9 insect cells afterbeing infected with recombinant baculoviruses synthesizing E2 as part of CE1E2: wild-type E2 (WT) and E2 with mutations of the glycosylation sites N1; N2; N10; N1–N11 (ΣN; mutations of all E2 glycosylation sites); WT+Ab CD81 and N1+Ab CD81, Huh-7 cells after pre-incubation with Ab CD81; C – negative control (Huh-7). Mutant proteins are marked as E2*

We have studied binding of HCV VLPs carrying mutant E2 proteins to Huh7.0 cells. Huh7.0 cells treated and untreated with specific anti-CD81 antibodies were incubated with VLPs derived from insect cells. After incubation with antibodies, binding of mutant E2 proteins to the cells was analyzed using Western blotting (*Fig. 9*). The working concentration of Ab CD81 was preliminarily determined (20 μg/ml) [26].

It was shown that HCV VLPs formed in insect cells and containing mutants of the E2 protein are bound to Huh7.0 cells regardless of whether the CD81 receptor is present on their surface or not. Helle*et al.* [26] have demonstrated that mutant E2 proteins as part of virus particles bind to HepG2, Huh7.0 cells in a CD81-dependent manner. It is understood that the influence of the CD81 receptor on the binding HCV virus particles to different cell types (HepG2, Huh7.0, NKNT-3, Molt- 4) manifests itself in different ways [27].

## CONCLUSIONS

Glycosylation of viral envelope proteins in an infected cell is the crucial stage in the morphogenesis of the hepatitis C virus, determining proper virion assembly. We have demonstrated that HCV envelope proteins synthesized in insect and mammalian cells, and in particular the E2 protein containing mutations at the glycosylation sites, are incorporated into the ER membranes, where their folding, formation of the E1E2 complex, and virus-like particles take place. Investigation of the role of the glycosylation of envelope proteins in the morphogenesis of the hepatitis C virus (genotype 1b strain 274933RU) revealed that disruption of the single glycosylation sites N1 and N8 of HCV protein E2 (as well as the N1 and N5 sites of HCV protein E1) enhances the expression of these proteins in mammalian cells in contrast to expression in insect cells. It was revealed for the first time that disruption of the N1, N2, and N10 glycosylation sites of the E2 protein (as well as the N1 and N5 sites of HCV protein E1) influences the formation of functional E1E2 heterodimers. Unproductive dimers are predominantly formed at these sites in the absence of glycans, while it does not impede the formation of HCV VLPs both in Sf9 insect and HEK293T human cells. We have put forward a hypothesis that the resulting virus-like particles with misfolded glycoproteins are defective and incapable of infecting target cells. These findings show that RNA-containing virus-like particles are formed with a density of 1.14–1.16 g/cm^3^ in Sf9 and HEK293T cells. It has been demonstrated that N-glycans of HCV glycoproteins have no effect on the synthesis of RNA structural proteins in insect and mammalian cells and perhaps on their incorporation into virus-like particles. We have shown that HCV virus-like particles synthesized in insect cells are bound to Huh7.0 hepatoma cells through the CD81-independent route.

## Abbreviations

CFU: colony forming units
HCV: hepatitis C virus
VLPs: virus-like particles
ER: endoplasmic reticulum
